# Language Lateralization in Children Aged 10 to 11 Years: A Combined fMRI and Dichotic Listening Study

**DOI:** 10.1371/journal.pone.0051872

**Published:** 2012-12-20

**Authors:** Fritjof Norrelgen, Anders Lilja, Martin Ingvar, Jens Gisselgård, Peter Fransson

**Affiliations:** 1 Department of Clinical Neuroscience, Karolinska Institutet, Stockholm, Sweden; 2 Department of Speech and Language Pathology, Karolinska University Hospital, Stockholm, Sweden; 3 Department of Neuroradiology, Karolinska University Hospital, Stockholm, Sweden; 4 Psychiatric Division, Regional Centre for Clinical Research in Psychosis, Stavanger University Hospital, Stavanger, Norway; University of Manchester, United Kingdom

## Abstract

**Objective:**

The aims of this study were to develop and assess a method to map language networks in children with two auditory fMRI protocols in combination with a dichotic listening task (DL). The method is intended for pediatric patients prior to epilepsy surgery. To evaluate the potential clinical usefulness of the method we first wanted to assess data from a group of healthy children.

**Methods:**

In a first step language test materials were developed, intended for subsequent implementation in fMRI protocols. An evaluation of this material was done in 30 children with typical development, 10 from the 1^st^, 4^th^ and the 7^th^ grade, respectively. The language test material was then adapted and implemented in two fMRI protocols intended to target frontal and posterior language networks. In a second step language lateralization was assessed in 17 typical 10–11 year olds with fMRI and DL. To reach a conclusion about language lateralization, firstly, quantitative analyses of the index data from the two fMRI tasks and the index data from the DL task were done separately. In a second step a set of criteria were applied to these results to reach a conclusion about language lateralization. The steps of these analyses are described in detail.

**Results:**

The behavioral assessment of the language test material showed that it was well suited for typical children. The results of the language lateralization assessments, based on fMRI data and DL data, showed that for 15 of the 17 subjects (88%) a conclusion could be reached about hemispheric language dominance. In 2 cases (12%) DL provided critical data.

**Conclusions:**

The employment of DL combined with language mapping using fMRI for assessing hemispheric language dominance is novel and it was deemed valuable since it provided additional information compared to the results gained from each method individually.

## Introduction

For patients with medically intractable epilepsy the option of epilepsy surgery is considered if the origin of the seizures can be located. In the presurgical planning of such cases it is important to determine hemispheric dominance for language to weigh possible benefits of the surgery against the risks of postoperative deficits. Previously the intracarotid amobarbital test, IAT (also known as the Wada test) was the state of the art method for determining hemispheric language dominance [1] but it is also invasive, known to carry risks for complications [2], and is more expensive than language mapping using fMRI [3]. In a number of studies results from WADA testing and fMRI have been compared and in spite of considerable methodological differences between studies an overall concordance between these two methods of approximately 90% has been reported [4], [5], [6]. Intra-subject reproducibility of global and regional language lateralization results with fMRI within and across sessions for epilepsy patients has been reported to have sufficient reproducibility for clinical use [7], [8], [9]. Language fMRI is now being used in many centres as a clinical tool in the planning of neurosurgical treatment of epilepsy in adults and, increasingly, in pediatric populations [3], [10].

Functional MRI is sensitive to subject motion, motivation and a good compliance to the tasks to be performed inside the MR-scanner. Therefore good preparation and training prior to assessment is critical, particularly with children. A large study of 209 children between 5 to 18 years of age reported an overall success rate of approximately 80%, with age being an important factor [11]. From one centre it is reported that with thorough preparation and training one can expect to obtain reliable and useful data in 95% of typically developing children aged 8 and older and in 80% of typically developing children aged 4 to 5 years old [12]. Gaillard and colleagues report high success rates with older children and early teenagers with neurodevelopmental disorders which are important aspects since such disorders are common in children with epilepsy [13].

A wide variety of language- and control-tasks have been used in studies mapping language regions in pediatric populations. Examples of paradigms that have been used are: semantic categorization of auditory presented words (animals that are both native to the United States and commonly used by humans, e.g. squirrel, yes/no) vs. tone-discrimination [14], verb-generation of nouns (pictures or written words) vs. visual fixation on a point [15], finding matching words for sentences (e.g. long yellow fruit –banana) vs. looking at row of dots [16], listening to short stories vs. listening to backward speech [17], semantic same-different discrimination of sentence-pairs vs. discrimination of same/different pairs of letter-strings [18], making decisions about semantic or syntactic correctness of read or heard sentences vs. deciding whether the patterns in rows of lines or two tones, respectively, are same/different [4].

It is well known that results from language-mapping fMRI are heavily influenced not only by the task used but also depend on the control condition employed. In a well-balanced fMRI paradigm the control condition should contain the same subcomponents as the task but not include the cognitive component to be examined, which has not always been the case in earlier studies [10].

It has been suggested that by using a panel of several language tasks the chances for successfully mapping language lateralization with fMRI in older children and adults improve [19], but in a clinical setting the full scope of such an approach is often not feasible due to limitations in the available time for each patient. Another important methodological consideration is that language fMRI data from individuals with lesions, which is common in epilepsy surgery patients, can be less reliable than in healthy individuals [18]. In a recent review of fMRI vs. WADA the authors argue that there may still not be sufficient data to support language fMRI as a routine procedure prior to epilepsy surgery [20]. These findings suggest that for some of the patients with intractable epilepsy it is not at all certain, or perhaps even likely, that adding more language fMRI tasks in an assessment would increase the reliability of the results. Therefore, an alternative route may be to provide an independent measurement by using a different method such as dichotic listening. Dichotic listening (DL) of two disparate consonant-vowel syllables presented to each ear has been used as a behavioral measure of language lateralization in adults and in children in a number of studies [21], [22], [23], [24], [25], [26]. Comparisons of language lateralization results from DL with WADA, PET and fMRI have shown that there is high correspondence between results and it has also been demonstrated that DL activates language networks [25], [27], [28], [29]. Taken together these results imply that DL may provide a good independent measure of language lateralization. To the best of our knowledge, no previous studies of language lateralization in children have combined language fMRI with DL. Hence, the aims of the current study were to develop and assess a methodology to map language networks in children that included two auditory fMRI protocols as well as a DL task. The proposed method is intended for assessments of pediatric patients prior to epilepsy surgery. In this study, however, we first wanted to assess a group of healthy children in order to evaluate the potential clinical usefulness of the method.

In the planning phase of the present study an important aspect that had to be addressed was the need for a test procedure for which the majority of the patients would be able to understand and perform well on with relatively little preparation. Since a considerable proportion of the pediatric population with pharmacologically resistant epilepsy can be expected to have reading difficulties [30] we decided to use auditory, rather than visual, stimulus presentation. We also argued that tasks with auditory stimulus presentation probably would simplify the training and therefore improve the subject’s ability to participate successfully. A consideration in the selection of language tasks was that they should primarily target the Broca- and the Wernicke-regions in the brain respectively. A task that previously has been shown to robustly activate the Broca region is verb generation [31]. Verb generation has also been used in children and it is relatively easy to train them to perform well [15]. Apparently, it has been shown to be more challenging to develop language tasks for children that consistently activate the Wernicke region [14]. Since temporal lobe epilepsy is very common in our target population – epilepsy surgery patients – an important aspect in the planning of our study was that our fMRI imaging protocol should also be able to map language areas in the temporal lobe [32]. An auditory language task that has been shown to activate the Wernicke area and that has been used successfully in children is story listening with the linguistic complexity being adapted to the subjects language level [17]. Both type of tasks, verb generation and story listening, are well suited for auditory stimulus presentation.

Another aspect that we considered in the planning of this study was that since the number of pediatric patients undergoing epilepsy surgery is small and they naturally cover a wide age range or range of language abilities, the best use of resources would be to develop test materials for verb generation and story listening that largely covered for this range in age or in language ability in the target group. In order to examine the extent to which the developed language material was suited for implementation in fMRI paradigms behavioral assessments of this material was also planned for. For the next step –the fMRI assessment– we considered the options of assessing a group with mixed age in correspondence to the developed language materials, or to assess a unified age group. A potential methodological problem with a mixed age group would be if the fMRI results would be heterogeneous, or difficult to interpret; we would then not be able to rule out developmental aspects as a confounder. It has been shown that there is some degree of developmental effects on language fMRI in children [33], [34] and therefore we decided to assess a group of a uniform age range.

In the current study we first present the steps taken to develop suitable language test materials for a verb generation and a story listening task, with the target pediatric population in mind. Moreover, we present the results from a behavioral assessment of the materials and the adaptations of them for implementation in two fMRI paradigms. Second, we present results with regard to language lateralization in pediatric subjects based on our approach that combined fMRI and DL data. Finally, we describe in detail how the results from our procedure were used in the process of reaching a conclusion about each subject’s language lateralization in the brain.

## Materials and Methods

### Ethics Statement

Signed consent was required of both parents of the children participating in this study. All examinations were carried out according to the ethical guidelines and declarations of the Declaration of Helsinki (1975) and the current study was approved by the regional ethics committee at the Stockholm County (2008/1826–31).

### Screening Tests

#### Tests of the language aptitude

A screening of language comprehension was performed with the subjects that participated in the behavioral assessment of the language test material and with the subjects that participated in the fMRI assessment. For this purpose two test were used; SPIQ [35] and TROG-2 [36]. SPIQ is a linguistic association test that examines the understanding of concepts and vocabulary (split-half reliability is 0.84). The task is to associate words with one of four pictures. For each item the test leader says the words loud and the child points to one picture. There are Swedish norms for this test that were used. TROG-2 tests the understanding of twenty grammatical constructs, each construct being tested four times using different test stimuli. For each test stimuli –a spoken sentence– the task is to point to one of four pictures that contain lexical and grammatical foils. The Swedish norms were used [37] (reliability measured by Cronbachs alpha is 0.89).

#### Handedness assessment

For the subjects participating in the fMRI assessment, the Edinburgh Handedness Inventory -revised, was administered to assess their hand preference [38].

### Development of Test Materials

#### Verb generation paradigm

In spite of the fact that verb generation tasks has been used in many fMRI studies we did not find any published developmental data on latencies for children for verb generation of nouns. This hiatus is surprising since different versions of this task has been used in a number of studies with language fMRI and latencies are clearly relevant for how the fMRI task is designed and obviously for the subjects ability to perform in the task. The assessments of three age groups (7–8, 10–11 and 13–14 years) would allow us to evaluate developmental aspects of verb generation, particularly regarding latencies, and inform us of potential necessary age-related adaptations of the material for implementation in fMRI paradigms. We decided to limit the latency for a single verb production to ≤2500 ms for fMRI implementation. This time limit was arbitrary but it was based on our intention to balance the number of trials with a reasonable length of the resulting fMRI sessions. For the verb generation task eighty common Swedish nouns, with a likely associated coupling to common Swedish verbs, were selected (e.g. scissor-cut, pen-write, and glass-drink). For the control condition in the verb generation fMRI paradigm we chose to use silent word repetition. For this purpose, 80 common Swedish adjectives were selected (e.g. red, big, and hot). The lists of verbs and adjectives were read by a trained native female Swedish speaker and recorded in a voice studio. In the next step two sets with 7 nouns and 7 adjectives in each set were selected from the corresponding lists of verbs and adjectives to be implemented in the verb generation paradigm; the nouns for the test condition and the adjectives for the control condition. To reduce the risk of any confusion about the task connected with each stimulus presentation during the fMRI sessions, a prompt was added before each stimulus (e.g. in test condition: –*what can one do with a pen*, and in control condition: –*repeat the word red*).

#### Listening paradigm

Since it has been shown that high cognitive load in language comprehension tasks may generate bilateral activation patterns [39], great care was taken to adapt the content of the test material to suit children with different language abilities. The age spans chosen were 7–8, 10–11 and 13–14 years of age. The process to develop the material was in short as follows. Suitable books for the corresponding age groups were selected on the basis of a combination of age recommendations in the databases at National Library of Sweden (http://libris.kb.se/), data from a national annual contest in which children of different ages score popular books (Barnboksjuryn: http://barnensbibliotek.se/), and recommendations of librarians working with children. From the selected books short passages were chosen that fulfilled the following criteria: (1) the text passages should be between 80–120 words long, (2) they should not contain uncommon words or complex or ambiguous sentences, (3) the content should not be emotionally upsetting and (4) the passages should be reasonably complete in themselves to prevent the listener from brooding about the continuation of the story. In some instances minor adaptations of words or sentences were made to fulfill these criteria. Thirty passages were selected, ten for each age group. The stories were read by a trained native female Swedish speaker and recorded in a voice studio.

A copy with reversed speech (i.e. played backwards) of each recorded passage was created to be used as a control condition in the fMRI paradigm. Reversed speech has been demonstrated to effectively remove semantic processing in similar tasks [17], [40]. The sound files were then compiled into two sets for each of the three age groups with each set containing five passages and five copies with reversed speech. These compilations were then implemented as epoch-related episodes in Presentation (Neurobehavioral Systems, www.neurobs.com). The mean lengths of the passages in the fMRI paradigms that were used in this study were 98.4 words (range 83–117) and 35.1 sec. (range 31–46).

### Participants

#### Recruitment of subjects for behavioral assessment of the language test material

Pupils in the first, fourth and seventh grades were recruited from a school in a socio-economically representative area of Stockholm. Teachers were requested to suggest three boys and three girls from each of their classes. The selection criteria were that the pupils should have Swedish as their native language and that their overall learning ability was considered to be on an average level, i.e. that they neither should be among the above average students nor that they should be considered to have any learning difficulties. The rationale for using these criteria was to improve the probability that the subjects were representative of typically developing children. The teachers sent the names of the pupils that they had selected to us and we then called the parents to inform them about the study. If the parents expressed interest to let their child participate in the study, detailed information directed to the child and to the parents was sent home so that they together could decide if they wanted to participate. Fifty pupils were contacted and 30 of them decided to participate in the study.

#### Recruitment of subjects for fMRI examination

As mentioned earlier language fMRI results may be influenced by development [33], [34] and to avoid this potential problem in the interpretation of the fMRI data we decided to assess a group of healthy children of similar age; 10–11 year-olds. The subjects were recruited from the same school, with the same selection criteria, and in the same manner as described in the previous section but from different classes. Thirty pupils were contacted and 18 of them consented to participate in the study. Due to technical problems during the MR-scanning procedure in one subject, the final number of subjects that underwent fMRI was 17. The gender distribution was 8 boys and 9 girls with a mean age of 10.6 years (SD 0.34).

### Procedures for Behavioral Assessment of Language Test Material

#### Verb generation material

The purpose of measuring the verb production latencies were 1) to identify nouns that produced too long verb generation latencies and which therefore were not appropriate to include in the fMRI material, and 2) to identify overall differences in verb generation latencies related to age that might be important for the design of the fMRI material. The criterion used for inclusion of nouns was set to a maximum latency of 2500 ms. Each subject was first given instructions about the task of overtly creating verbs from nouns. When it was clear that the subject had understood the task, a semi randomized list of the nouns was read aloud by the test leader, one by one, and the subject responded by producing a verb to each presented noun. These sessions were recorded for later analysis of latencies. The latencies were subsequently measured by manually determining the time interval between the end of each presented noun and the beginning of the produced verb from the recordings of each subject.

#### Listening material

The purpose of examining the intelligibility of the language material for each age group was to identify passages that were cognitively demanding in order that such passages could be excluded. High cognitive load in language tasks may generate bilateral cortical activations [39]. The recorded passages were presented one by one in a semi-randomized order. Following each passage, two specific questions were given about the content of the passage. Each response was scored as, 0 for incorrect response, 1 for a partly correct response, or 3 for correct response. The basis for using this scoring system is that having a gap between 1 and 3 points improve the differentiation between slightly deviant answers and completely correct responses [41]. The following is an example from the assessment with a question and examples of the scoring of responses: This particular story was about some children who went for a swim in a lake in early spring (one of the questions was –why did the adults say that the children were “young fools”?). Zero point: no answer or an irrelevant response, one point: “because it was cold” or “because they went swimming”, three points: “because they went swimming even though the water was very cold”. Thus, the responses to the two questions regarding each passage resulted in a total score between 0–6. Scores between 1–2 points (17–33%) indicate a poor understanding of the story, 3 points (50%) indicate a good understanding of part of the story and 4–6 points (67–100%) indicate a good understanding of the whole story. Four points meant that the child gave a precise answer to one of the two questions for each short story, and also provided some information on the second question, which together was interpreted as that the understanding of the whole story was good. Therefore 4 points was set as the criterion score for inclusion of the corresponding story in the listening paradigm for the fMRI assessment.

### Procedures for Assessments of Language Test Materials with fMRI

#### Preparation and pre-training for fMRI session

On arrival the subjects were first briefly guided around the MRI facilities and were then given a step-by-step preparation of the whole procedure in the MR-scanner. The preparation began with general information about the MR-scanner and the equipment and was followed by step-by-step instructions about the procedure. The step-by-step instructions consisted of verb-generation and word-repetition tasks, first overtly and then covertly (as in the scanner), concluded by performing a full mini-version of the verb generation paradigm. The language materials used in the pre-training did not include items that were used in the fMRI assessments. Following this, the subjects received instructions about the listening task. They were told that they should listen to the stories and to the sounds (the control task consisting of backward speech). The control task was not described as backward speech but as meaningless, speech-like sounds. They were also informed that they would receive questions about the content of the stories after the assessment in the scanner. Finally, they performed a recorded complete mini-version of the listening paradigm. The time needed for this whole preparation with each subject was 15–20 minutes. Immediately following this procedure they were brought to the MR-scanner for the fMRI sessions.

#### Image acquisition and fMRI paradigm design

A 1.5 Tesla GE (General Electric Healthcare, Milwaukee) HDxt scanner equipped with a quadrature Tr/Tx head coil was used. Anatomical MR imaging included a high-resolution spoiled gradient recalled 3D T1-weighted image sequence (TR/TE = 24/6 ms, flip = 35 deg, FOV = 220×220, matrix size 256×192, (0.75% phase FoV)) that provided whole brain coverage with a spatial resolution of 0.9×0.9×1.5 mm^3^ in a coronal slice orientation. Functional MRI image volumes of the brain were acquired using a gradient Echo-Planar Image (EPI) sequence (TR/TE = 2500/40 ms, flip = 90 deg, FOV = 220×220 mm, slice thickness = 4.5 mm, slice gap = 0.5 mm, matrix size = 64×64). Each EPI BOLD volume consisted of 32 contiguous axial slices with a spatial resolution of 3.44×3.44×5 mm^3^.

Both the listening task and the verb generation task were implemented as block-related fMRI designs. The listening task consisted of epochs of speech of approximately 35 seconds in length interleaved with periods of reversed speech of equal length. The listening task was divided into two separate fMRI session that each entailed four epochs of speech mixed with five epochs of backward speech (i.e. each session starting and ending with a reversed speech epoch).

Similarly, the verb generation task consisted of two separate fMRI sessions, for which each session contained four epochs of verb generation and five of word repetition, respectively. The total MRI scanning time was approximately 45 minutes. In each subject, 150+134 EPI image volumes were acquired during the listening tasks and 126+126 EPI volumes were obtained for the verb generation task.

#### Image analysis

All pre-processing and statistical analysis were carried out in SPM5 [42]. Initially, all EPI volumes were spatially realigned and corrected for movement and subsequently normalized to the MNI (Montreal Neurological Institute) EPI template within SPM and re-sampled to 2×2×2 mm3 voxel size. Finally, normalized EPI volumes were smoothed using a spatial filter kernel of FWH = 8 mm. BOLD signal increases pertaining to task-evoked responses in brain activity related to passive listening of speech versus backward speech, as well as verb generation versus word repetition, respectively, were modelled using a general linear model (GLM) as implemented in SPM. Six regressors modelling residual movement related variance (translational and rotational movement) were included in the model as covariates of no-interest. At the individual level, statistical parametrical maps showing brain activation related to verb generation and passive listening were thresholded at p<0.001 uncorrected. In this context, the usage of an uncorrected statistical threshold is warranted due to the fact that we were primarily interested in brain activation patterns at the individual level. We believe that the thresholding level chosen here provides a good compromise between the risk of type-I versus type-II errors in the statistical analysis of fMRI data, in particular when the focus of investigation is to study brain activity at the subject level. This is also in agreement with previous fMRI studies of brain laterality of language function pediatric populations [15], [17], [33], [43]. Language lateralization was quantified using the index as implemented in the Lateralization Index (LI) SPM toolbox [44], values were computed separately in the frontal, temporal and parietal lobe. Positive values indicate a left-sided hemispheric dominance and negative values indicate a right-sided hemispheric dominance.

#### Dichotic listening test

The dichotic listening test [24] (DL) consists of consonant-vowel syllables presented pair-wise to both ears simultaneously via a headset (consonants: p, t, k, b, d, g, and vowels: i, a, u). During each trial two different consonant-vowel syllables are presented, one to each ear (e.g. da-bi). Each stimulus presentation is approximately 350 ms with an inter stimulus interval of 4 sec. The subjects were instructed that the sounds are not “real words”, that they “do not mean anything”, and that they should say out loud both of the two syllables, or one of them if they only managed to perceive one. One test session involved 108 stimulus pair presentations. The subject’s responses were manually registered by the test leader on a computer. A laterality index for consonants and vowels was calculated (right ear minus left ear scores of correctly perceived consonants and vowels, respectively, divided by right ear plus left ear scores ×100). Right ear advantage (REA) was defined as a laterality index equal to or greater than 5 and left ear advantage (LEA) when it was equal to or below −5, no ear advantage (NEA) for indices between 5 and −5 [45]. REA indicate a left-sided hemispheric dominance and LEA indicate a right-sided hemispheric dominance.

#### Behavioral assessments

Immediately after MR scanning, a semi-structured interview with the subjects followed with questions about the content of the stories in the listening paradigm and about their own impression of participation. The purpose of the interview was to get an indication of their participation during the fMRI sessions. This was followed by an audiometric screening test (125–8000 Hz at 20 dbl) and the DL test. The two language aptitude tests were then administered assessing vocabulary (SPIQ) [35] and language comprehension (TROG-2) [36] and finally, in order to confirm that all children were right-handed, handedness was assessed by the Edinburgh Handedness Inventory, revised [38].

### Procedure for Determining Hemispheric Language Dominance

The procedure to come to a conclusion about hemispheric language dominance consisted of several steps; fMRI data and DL data were analyzed separately to determine whether the result from each test was conclusive or not. The results of these two assessments were then analyzed in order to reach a conclusion regarding hemispheric language dominance. The criteria employed in these procedures are specified in detail in Table 1. One aspect that had relevance for the design of the criteria for the evaluation of fMRI data was that we considered activations in the frontal lobe on the verb generation task and activations in the temporal lobe on the listening task to have a higher value than activations in other regions. The basis for this assumption is that the most consistently found activations of verb generation tasks are in the inferior part of the frontal lobe [46] and activations in this region were therefore considered to be highly dependable. Similarly, the listening task has been demonstrated to specifically activate the temporal lobe [17].

**Table 1:** The criteria applied in the analysis of the fMRI data (1.), and of the dichotic listening data (2.), and the criteria used to combine these two analyses to reach a final conclusion about hemispheric language dominance (3.).

**1. fMRI-results on verb generation task and listening task (the applied index threshold value for regional hemispheric dominance ≥ ±0.2)**
Conclusive result:
1a) Two or more consistent index values in one task alone or in both tasks together, and no contradictory index value(s).
1b) Result as 1a, and with an additional contradictory index value not in the target region of the corresponding task (target region is the frontal lobe in the verb generation task (46) and the temporal lobe in the listening task (17).
Inconclusive result:
1c) No index value.
1d) Only one index value.
1e) Two single contradictory index values in one paradigm or between paradigms.
1f) Result as in 1a, and with an additional contradictory index value located in the target region of the corresponding paradigm.
1g) Result as in 1f but with 2≥ contradictory index values regardless of target region(s).
**2. Dichotic listening results (the applied index threshold value for hemispheric dominance ≥ ±5.0)**
Conclusive result:
2a) One index value.
2b) Two consistent index values.
Inconclusive result:
2c) No index value or two contradictory index values.
**3. Weighted results from fMRI and dichotic listening (DL) for the assessment of language lateralization**
Conclusive result:
3a) Conclusive fMRI result alone (1a or 1b), or together with a consistent result from DL (2a or 2b).
3b) Conclusive fMRI result (1a, but not 1b) in combination with a contradictory or inconclusive DL result (2a or 2c but not 2b).
3c) Inconclusive fMRI result (1d or 1e) with an index value in the target region for the corresponding paradigm consistent with the DL result (2b).
Inconclusive result:
3d) Inconclusive fMRI (1c, 1f or 1g) regardless of DL result (2a, 2b or 2c).
3e) fMRI result as in 1b but contradicted by one or two indices in DL.

## Results

### Results from Behavioral Assessments of the Language Test Materials

Prior to implementing the language test material for use with fMRI, behavioral assessments on a group of typical children were made. In Table 2 the group composition, the results on the language screening tests, as well as the results from the listening- and verb generation tasks, are displayed for each school grade. The scores on the vocabulary test (SPIQ) were within the normal range for 29/30 subjects (between the 15^th^ and the 85^th^ percentile) and the scores on the language comprehension test (TROG-2) were within the normal range for all subjects^*^. The overall results of these two tests indicate that the language development of the subjects was representative of typically developing children.

The individual that performed below the 15^th^ percentile on the vocabulary test in contrast performed above average on TROG-2 (standard score 113). His results on comprehension of the stories in the listening task and the latencies in the verb generation task were also within the average of the whole group. The results indicate that this individual can be considered as representing a typical language development.

**Table 2:** Group composition and means and standard deviations on the language screening tests –vocabulary and language comprehension– and on the listening- and verb generation tasks.

School Grade	Gender	Mean age	Vocabulary test:SPIQ (staninepoints)	Language comprehension test: TROG-2 (standard scores)	Listening task: comprehension (average percent correct/passage)	Verb generation: latencies (ms)
**One (n 10)**	6 f, 4 m	7.71 (0.24)	4.30 (1.49)	113.90 (5.47)	87.17 (12.98)	1546.55 (424.09)
**Four (n 10)**	6 f, 4 m	10.83 (0.39)	5.40 (1.08)	104.50 (2.84)	89.17 (9.14)	1229.77 (487.46)
**Seven (n 10)**	5 f, 5 m	13.66 (0.47)	3.50 (0.71)	104.20 (5.12)	87.83 (10.06)	1194.79 (459.87)

The overall mean scores of the comprehension of the stories in the listening task were high for all three age groups (see Table 2). The lowest mean score for comprehension of any of the stories was 4.3 (72%). The lowest overall mean score of the stories at the individual level was 3.9 (65%). This particular subject scored normally on the language screening tests and was at level with the other subjects on the latency on the verb generation task. The results of the assessments of comprehension of the stories was high for all three age groups, indicating that these stories were well suited to be included in the listening paradigm for the fMRI assessment.

The overall mean results of the latencies in the verb generation task are displayed in Table 2. A criteria for the usefulness of the nouns in the fMRI paradigm was a maximum latency of 2500 ms. The latencies for the produced verbs derived from two of the nouns in the list exceeded this limit and these nouns were therefore excluded. An additional noun (mouth) was removed because it obviously caused embarrassing associations for several of the subjects. Analyses of variance of the results showed that there was a weak trend of first graders having longer latencies than the fourth and seventh graders but that this difference was not significant [F(2,27) = 1.791, p = 0.19]. There was also a non-significant overall trend of girls having shorter latencies than boys in this task [F(1,28) = 1.643, p = 0.21]. There were no significant overall differences between the results for boys and girls on the tests of vocabulary [F(1,28) = 0.003, p = 0.96], language comprehension [F(1,28) = 0.030, p = 0.86] and on the listening task [F(1,28) = 0.113, p = 0.74]. It should be added that the verb generation task was completely unproblematic for all of the subjects to perform.

### Results from Behavioral Assessments of the 10–11 Year Olds Assessed with fMRI

All subjects were right-handed (mean handedness index of 97, range 77.8–100). The group mean standard score on the screening test of language comprehension (TROG-2) was 97.24 (8.09). On the test of vocabulary the group mean stanine score was 5.82 (1.78). The mean results for all individual subjects were within the normal range. All subjects passed the audiometric screening test.

### fMRI Results

#### Whole brain analyses

**Figure 1:**
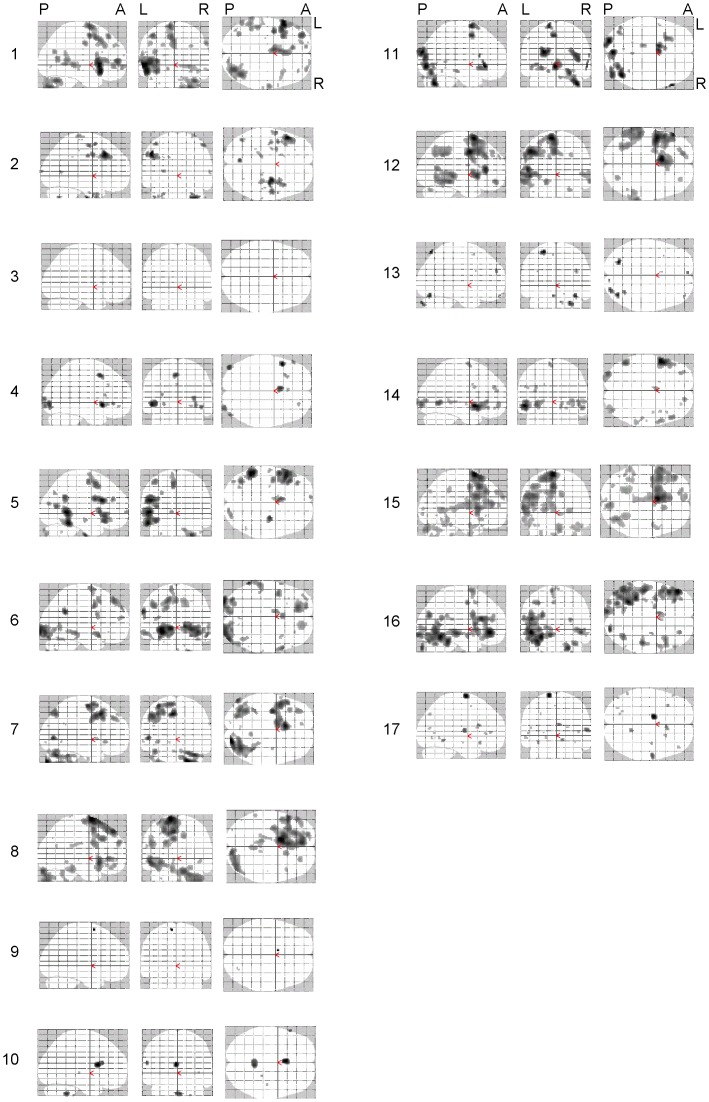
Maximum Intensity Projections (MIP) of fMRI data for subject 1 to 17 in the verb-generation paradigm at p<0.001 uncorrected. Spatial orientation of all projections is shown on the top row (P = posterior, A = anterior, L = left, R = right).

**Figure 2:**
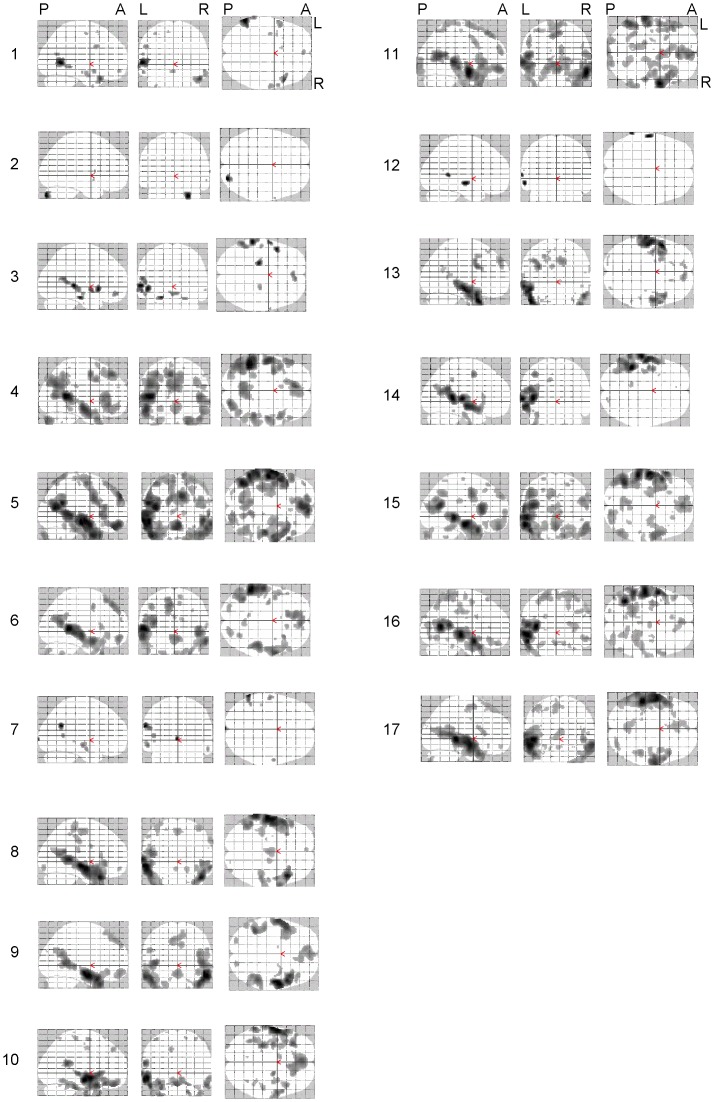
Maximum Intensity Projections (MIP) of fMRI data for subject 1 to 17 in the listening paradigm at p<0.001 uncorrected. Spatial orientation of all projections is shown on the top row (P = posterior, A = anterior, L = left, R = right).

The whole brain analyses on the verb generation task and on the listening task were based on significant BOLD signal changes in response to the task condition contrasted by the control condition (thresholded at p<0.001). The results are presented as maximum intensity projection maps (Figures 1 and 2 respectively).

**Figure 3:**
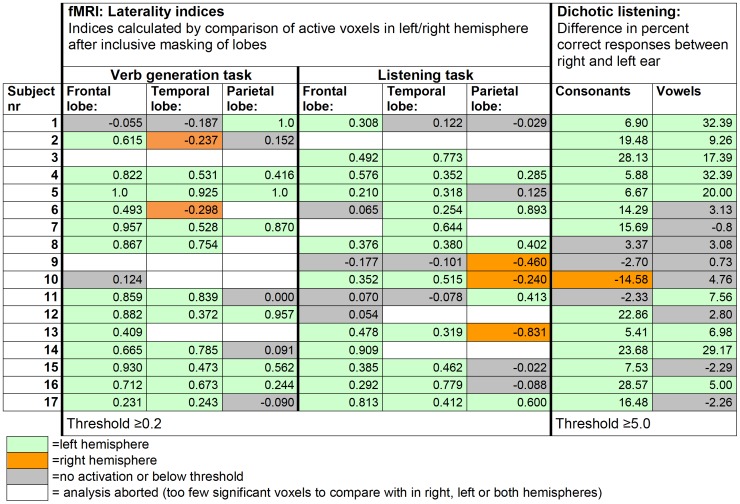
Results on the two language fMRI tasks and on dichotic listening for each subject. The fMRI results on each task are based on significant BOLD signal changes in response to the task condition contrasted by the control condition.

#### Laterality index analyses

Regional hemispheric dominance was considered as present when Laterality indices (LI) were≥±0.2 [14], [47]. We analyzed the results from both the verb generation paradigm and from the listening paradigm in accordance with the criteria (see Table 1) to reach a conclusion about the overall result of the fMRI assessment (see Figure 3). Fifteen subjects had a conclusive result (88%); twelve fulfilled criterion 1a (nr 1, 3, 4, 5, 7, 8, 11, 12 and 14–17) and three subjects fulfilled criterion 1b (nr 6, 10 and 13). Two subjects had an inconclusive result (12%); one fulfilled criterion 1e (nr 2) and one subject fulfilled criterion 1d (nr 9).

#### Dichotic listening

The analyses of the DL data revealed that the results for 15/17 (88%) of the subjects were conclusive (see Figure 3). Fourteen (82%) subjects displayed results above threshold on discrimination of consonants and nine (53%) subjects on discrimination of vowels. The results on consonant- and vowel discrimination were not discrepant for any subject. All results pointed to left-sided hemispheric dominance except in one case (subject nr 10).

#### Hemispheric dominance

In order to reach a conclusion about hemispheric dominance we considered the results from the laterality index analyses and the results on the DL test taken together according to the specified criteria 3a–3e (Table 1). Fifteen subjects (88%) had a conclusive overall result; thirteen subjects fulfilled criterion 3a (nr 1, 3–7 and 11–17), one subject fulfilled criterion 3b (nr 8) and one subject fulfilled criterion 3c (nr 2). All subjects displayed a left sided hemispheric language dominance. The results of two subjects were inconclusive; one fulfilled criterion 3d (nr 9) and another subject fulfilled criterion 3e (nr 10). In these analyses DL provided critical information in two cases (nr 2 and 10).

## Discussion

In this study two different language fMRI paradigms using auditory stimulus presentation were used in combination with a DL test to assess language lateralization in seventeen typical ten-year-old children. For 15 of the 17 subjects (88%) a conclusion regarding hemispheric language dominance was accomplished. The proportion of conclusive results about language lateralization in this study is comparable with those reported in other studies of language fMRI with typical children [11], [17], [19], [48]. In one of those studies [17] Ahmad and colleagues used a very similar listening paradigm as the one used in the present study to assess 15 children (mean age 6∶8). A comparison between studies reveal similar results of the proportions and regional distribution of activations above threshold in the listening paradigm; in Ahmad’s study 73% displayed activations in the temporal lobe and/or in the inferior parietal lobe (65% in our study) and 53% displayed activations in the middle or inferior frontal gyrus (67% in our study). They concluded that their results were promising for using the listening paradigm for targeting temporal language regions in language fMRI with children, which is supported by our findings.

In clinical language-fMRI the reliability of the result is critical and particularly one wants to avoid the risk of obtaining false hemispheric language dominance results. There is a broad consensus that results of language fMRI in general provide reliable results [7], [8], [9]. There are a number of studies investigating the overlap of results of language fMRI and results from WADA testing but little available data in the literature of the actual risk of obtaining false positive results with clinical language fMRI. The main reason for this being that there is no highly reliable measure of language lateralization that could be used in clinical practice to compare lateralization data from fMRI with. Previously, results from WADA testing were considered to provide such a stable and reliable measure with regard to brain lateralization, but this view has come to change over time and in a recent review it is concluded that fMRI generally provides higher accuracy and more detailed data than the WADA test does [49]. In our study we carefully considered and took measures to minimize the influence from experimental factors that could potentially have a strong negative impact on the reliability of the results and particularly against the potential problem of claims of language laterality that are based on a single parameter alone. We used two fMRI paradigms, tailored to engage different types of language processing (language comprehension and production of verbs), targeting different sub-components of the language network in the human brain. The fMRI paradigms described here were also conservatively designed in the sense that we strived to employ control conditions that minimized confounds from non-language related sources of brain activity. In addition, we employed identical statistical thresholding across all subjects in the fMRI analysis. Importantly, we used a DL task to provide an independent reliable behavioral measure of hemispheric dominance of language processing [45]. In two cases (12%) DL provided critical data. It is quite likely that this proportion would be higher in a clinical population with patients with intractable epilepsy.

In previous studies different language paradigms, as well as different strategies to process, compile and interpret data from language fMRI, has been employed. To date, no golden standard exist for how data from two or more different language fMRI tasks together with data from another modality (i.e. dichotic listening tasks) should be compiled and interpreted. However, as mentioned earlier, there is general agreement that results from language fMRI and dichotic listening, respectively, has high reliability. In this study a conclusion regarding language lateralization in each individual was reached based on an analysis of a compilation of lateralization indices for frontal-, temporal- and parietal lobes and the results from DL. We argue that the risk of obtaining incorrect results is substantially reduced by this combined approach. In those cases when the results contain ambiguous data, as was the case for three of the subjects in our study group (nr 2, 6 and 13), we believe that for clinical application a reassessment with fMRI and/or DL should be considered to obtain more reliable results. For subjects presenting with plainly contradictory results, such as the two subjects with inconclusive results in our study group (nr 9 and 10), and when there is no indication that the ability to participate in the fMRI tasks was poor, we believe it is most uncertain that an additional assessment would provide useful information.

Prior to implementation in the fMRI protocols the language material had been evaluated with typical children, a step which to our knowledge has not been taken in any previous study of language fMRI with children. The results of the evaluation showed that the stories from the listening material were well understood by the subjects, that the verb generation task was unproblematic for them to perform and that the latencies were well within the time frame for usage in an fMRI paradigm. Of importance in any kind of functional assessment of children using fMRI is to thoroughly prepare the subjects for the assessment to optimize their ability to cooperate well [12]. In this study we used a well-structured preparation of the subjects prior to the fMRI examination and we believe that this indeed was an important factor for the relatively high success rate, as has also been reported by others [12]. Neither during the pre-training of the subjects nor in the post-test interviews with the children after the fMRI sessions, was there any indication of difficulties to understand or perform the tasks involved in the fMRI examination.

A limitation of the present study is that the behavioral assessments, prior to fMRI, involved three different age groups whereas the assessments with fMRI and DL involved only 10-11-year olds. Thus it is uncertain if the validity of the present results can, without further investigation, be extended to also include younger and older subjects. The rationale for our decision to limit the assessments to children of one age group was to exclude age as a confounder in order to simplify the interpretation of the results. All previous similar studies have involved cohorts of children of a substantially wider age span.

Another caveat is that the fMRI paradigms used did not involve any objective measure of each subject’s performance of the language tasks during MR-scanning. There certainly are means by which this would be possible to achieve. However, that would not have been in line with our intentions of decreasing preparation time with each subject and more importantly to simplify the tasks in order to optimize the chances of good participation.

The DL task was used as a behavioral measure of language lateralization in our assessments. Fifteen of the seventeen subjects displayed results above threshold. For one subject (nr 10) the results were contradictory in relation to the visual fMRI data (that subject also displayed contradictory LI data and the overall result was inconclusive). The DL task is not time consuming and is relatively easy to distribute. For all subjects in this study it was unproblematic to participate in this assessment and from our clinical experience with epilepsy patients we would expect that the majority of such patients who would be capable to participate in an fMRI assessment would also be able to participate in a DL test. Our results suggest that DL is a useful behavioral complement to language fMRI data in assessments of language lateralization.

In summary, our results suggest that the methodology and experimental design used in this study is suitable for obtaining data with regard to language lateralization in typical children. We plan to proceed and investigate the usefulness of the proposed method in a clinical population of children who are candidates for epilepsy surgery.
